# The National Hospice and Palliative Care registry in Korea

**DOI:** 10.4178/epih.e2022079

**Published:** 2022-09-21

**Authors:** Kyuwoong Kim, Bohyun Park, Bonju Gu, Eun Jeong Nam, Sue Hyun Kye, Jin Young Choi

**Affiliations:** National Hospice Center, National Cancer Control Institute, National Cancer Center, Goyang, Korea

**Keywords:** Hospices, Palliative care, Registries, Statistics, Neoplasms

## Abstract

The National Hospice and Palliative Care (NHPC) registry is a nationwide database in Korea that systematically collects information on terminally ill cancer patients receiving inpatient hospice care. From 2018 to 2020, a total of 47,911 patients were enrolled in the NHPC registry from hospitals providing inpatient hospice care. The NHPC database mainly contains the socio-demographic and clinical information of the registered patients. Among these patients, approximately 75% were 60 years or older, and the ratio of males to females was 1:1.41. Lung, liver, colorectal, pancreatic, and gastric cancer made up nearly 90% of the cancer sites among the registered patients. Upon their initial admission to the hospice ward, around 80% of the patients were aware of their terminal illness. About half of the patients had mild pain at the time of the initial admission to the hospice ward, and the duration of hospice care was 14 days (interquartile range, 6-30) in 2019 and 2020. The NHPC registry aims to provide national statistics on inpatient hospice care to assist health policy-making.

## INTRODUCTION

Cancer is consistently among the major leading causes of death in Korea and elsewhere in the world despite some variations across countries by income level [1-3]. Compared to the previous year, cancer mortality increased by 1.2% in Korea in 2019, and cancer remained the leading cause of death, followed by cerebrovascular accidents [4,5]. However, there is a paucity of data on the most up-to-date national statistics on hospice and palliative care (HPC) in the previous literature. Among those who died from cancer, approximately 15% were reported to have been newly admitted to hospitals to receive inpatient hospice care at the end-of-life (EoL) in Korea in 2015 [6]. A previous study [7] describing terminally ill cancer patients receiving inpatient hospice care registered in the Korean Terminal Cancer Patient Information System (KTCPIS) was conducted in 2009. In addition to the outdated information describing the status of HPC, the KTCPIS is no longer in use for registering terminally ill cancer patients. Furthermore, the Act on Decisions on Life-sustaining Treatment for Patients in Hospice and Palliative Care or at the End of Life was enacted and enforced in 2016 and 2017, respectively [8]. In accordance with the Act, the National Hospice Center (NHC) was designated by the Ministry of Health and Welfare to collect, analyze, and provide statistics on the status of HPC in Korea [9].

This study aimed to describe the most up-to-date data collection procedure and components of the database derived from the National Hospice and Palliative Care (NHPC) registry, which is used to provide nationwide statistics on HPC in Korea.

## DATA RESOURCE

### The National Hospice and Palliative Care database

The NHPC registry was constructed to collect information and provide national statistics on patients at the EoL receiving HPC in Korea. The NHC at the National Cancer Center has been managing the NHPC registration system and database since 2017 and has published annual reports of HPC statistics. The NHPC registration system undergoes information system management and improvement on a regular basis to integrate the data collected from nearly 100 hospitals designated by the Ministry of Health and Welfare to provide HPC to terminally ill cancer patients. In accordance with Article 21 of the Act on Decisions on Life-sustaining Treatment for Patients in Hospice and Palliative Care or at the End of Life, the NHC is able to construct a nationwide database related to HPC among patients at the EoL.

## MEASURES

### The National Hospice and Palliative Care Study

#### Structure of the dataset

The currently operating NHPC registration system collects data on the terminally ill cancer patients using a common case report form (CRF) due to large variations in the data structure of the electronic medical record (EMR) system in each hospital that provides HPC. Prior to the implementation of the NHPC system, the CRF was both internally and externally reviewed by experts. To obtain and abstract data on socio-demographic factors, the initial assessment (i.e., clinical evaluation) of the patients, and death records, each hospital is required to review the EMR and submit appropriate data to the NHPC registration system on a regular basis. The cancer site of each patient was classified according to the Korean Standard Classification of Diseases-6 (KCD-6), which is comparable to the International Classification of Diseases, 10th revision (ICD-10). As most terminally ill cancer patients need extensive care, information on the primary caregiver, such as the legal relationship and whether the patient was living together with the caregiver prior to hospitalization, was collected. The key variables of the NHPC database are presented in Table 1.

#### Selection of the enrollees

Among the hospitals that specialize in providing inpatient hospice care, terminally ill cancer patients who were newly registered and began to receive HPC were enrolled in the NHPC registration system between 2018 and 2020. The assessment of terminal illness was determined by the physician in charge as well as the specialist in the relevant field according to clinical symptoms, the presence of other diseases or disorders, the degree of improvement due to drug administration or procedures, the progress of previous medical treatment, and availability of other treatment options for the patient. A total of 15,859 patients were enrolled in 2018, and 16,798 were enrolled in 2019, which was an increase of 5.9% compared to the year before. In 2020, the number of patients enrolled in the system was similar to that of 2018 (n= 15,254) (Table 2). No probability sampling method was necessary in the process of selecting enrollees because the NHPC registration system included virtually all terminally ill cancer patients receiving HPC in Korea.

### Data collection

**Figure 1.**
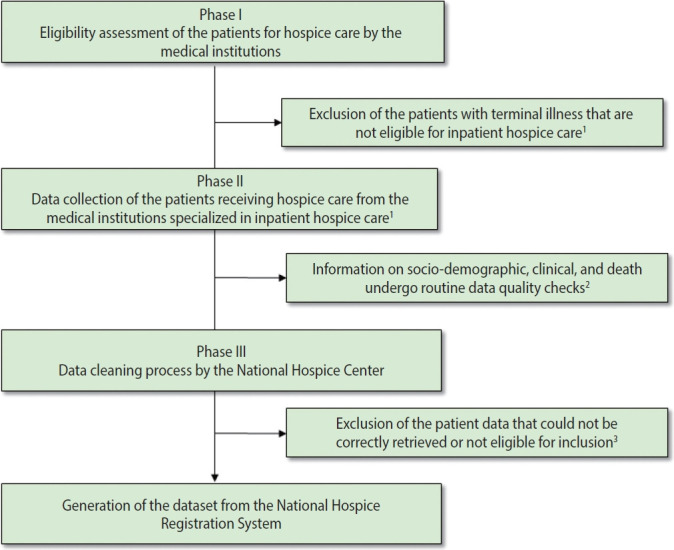
Flowchart of the data collection process for the National Hospice Registration System in Korea, 2018-2020. ^1^In accordance with the Act on Decisions on Life-sustaining Treatment for Patients in Hospice and Palliative Care or at the End of Life, only terminally ill cancer patients are eligible for inpatient hospice care. ^2^The National Hospice Center requests the correction of suspicious values entered by hospitals providing hospice and palliative care. ^3^Hospitals that were revoked of their designation status (medical institution specialized in hospice and palliative care) or closed (temporarily or permanently).

Currently, only terminally ill cancer patients are eligible for inpatient hospice care in Korea under the Article 2 of the Act on Decisions on Life-sustaining Treatment for Patients in Hospice and Palliative Care or at the End of Life. Accordingly, data on cancer patients who have limited life expectancy (i.e., without any possibility of recovery and who are expected to die within a few months) confirmed by medical doctors are collected through the NHPC registry. Prior to generating the final dataset used for producing national statistics on HPC, the collected data in the NHPC undergo data quality checks from the initial eligibility assessment to data cleaning (Figure 1). Employees of the NHC who are in charge of data collection routinely identify data in the NHPC registration system that might have been incorrectly entered based on the EMR from each hospital. From 2018 to 2020, there were around 100 hospitals from which nationwide data on HPC were collected (98 hospitals with a total of 1,542 hospice beds in 2018, 100 hospitals with a total of 1,577 beds in 2019, and 97 hospitals with a total of 1,546 beds in 2020, respectively). If a hospital providing inpatient hospice care was revoked of its designation status (i.e., as a specialized medical institution providing inpatient hospice care) by the Ministry of Health and Welfare or temporarily closed (i.e., due to the coronavirus disease 2019 pandemic or other reasons) data from that hospital were not included in the NHPC registry. In addition, data with suspicious values that could not be correctly retrieved by contacting the person in charge at each hospital were also excluded as of the last calendar date in each year. Thus, the NHPC registry only contains quality-assured nationwide data on inpatient hospice care.

### Ethics statement

The study protocol for the NHPC registry was approved by the Institutional Review Board at the National Cancer Center, Goyang, Korea (IRB No. NCCNCS09234). Written informed consent was obtained from each patient or his or her legal guardian upon registration. Administrative permission to access the NHPC registry was only granted to authorized personnel who were approved by the appropriate ethics committee of each hospital. The study protocol adhered to the legal regulations of Korea and was also in compliance with the Declaration of Helsinki.

## DATA RESOURCE USE

### Socio-demographic characteristics of the enrollees

From January 1, 2018 to December 31, 2020, a total of 47,911 terminally ill cancer patients were enrolled at 97 hospitals providing inpatient hospice care (as of 2020) in Korea. The socio-demographic characteristics of the newly registered patients receiving inpatient hospice care from 2018 to 2020 are shown in Table 3. Most patients (≥ 75%) were aged 60 years or more, and approximately 58% of the patients were male. More than two-thirds of the patients resided in the capital area or metropolitan area, and nearly 90% of them were covered by the National Health Insurance Service (i.e., employee-insured and self-employed insured), whereas around 9% of them were recipients of the Medical Aid program. Nearly 65% of the patients were married, and approximately 30% of them were either bereaved of their spouses or divorced at the time of enrollment. Fewer than 5% of the patients were single (i.e., not legally married) or separated (i.e., living separately regardless of the marital status). Among the patients who were reported to be religious, Protestantism was the most common religion (24.6 to 26.7%) followed by Buddhism (17.0 to 17.9%) and Catholicism (12.1 to 13.5%). Around 40% of the patients reported that they had no religious background at all. Family caregiving was the most common practice among the patients (92.2 to 95.5%). The distribution of patients’ socio-demographic characteristics remained consistent throughout the years.

### Clinical characteristics of the enrollees

Table 4 demonstrates the clinical characteristics of the patients enrolled in inpatient hospice care between 2018 and 2020. Among these patients, nearly 90% of the cancer sites were non-sex-specific. Lung cancer (ICD-10: C34) was the most common type of cancer among the terminally ill cancer patients (15.7-18.7%), followed by liver cancer (ICD-10: C22; 10.6-11.7%) and colorectal cancer (ICD-10: C18-C20; 10.3-11.5%), pancreatic cancer (ICD-10: C25; 9.4-11.0%), and gastric cancer (ICD-10: C16; 8.8-10.1%). Nearly 80% of the patients were aware of their medical condition (i.e., of the fact that they had terminal cancer that could not be cured or treated). This proportion steadily increased from 2018 to 2020 (81.6% in 2018, 82.9% in 2019, and 85.5% in 2020). The level of consciousness among the patients at the time of initial assessment was fairly consistent throughout the years. Most (≥ 70%) of the patients were alert when they were first admitted to the hospice ward, and nearly a quarter of them were drowsy (18.3 to 20.6%) or stuporous (5.7 to 6.5%). Fewer than 1% of them were in a coma upon admission (0.7% in 2019 and 0.8% in 2018 and 2020, respectively). About half of the patients had mild pain at the time of initial registration in the inpatient hospice ward on a scale of 0 to 10 (i.e., 0: none, 1-3: mild, 4-6: moderate, and 7-10: severe). The median duration of inpatient hospice care (i.e., from the initial admission to discharge) was 15 days (interquartile range [IQR], 6-31) in 2018 and 14 days (IQR, 6-30) in 2019 and 2020, respectively.

### National hospice and palliative care statistics

**Figure 2.**
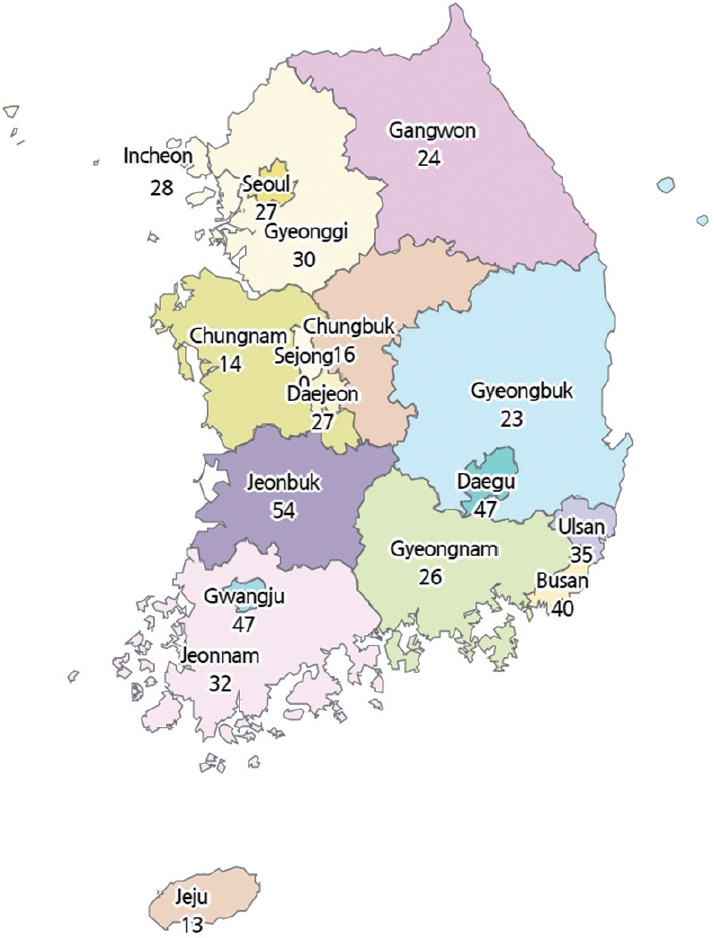
Number of hospital beds for hospice and palliative care per 1,000,000 people by administrative districts in Korea in 2020. The data presented show the number of hospital beds assigned for hospice and palliative care per 1,000,000 people in each administrative district in Korea in 2020 (Sejong has 0 beds for inpatient hospice care because it contains no hospital specialized in hospice and palliative care).

Currently, the NHPC registry is mostly used for producing national statistics on HPC in Korea. Notably, the hospice utilization rate is calculated by the number of newly registered patients receiving HPC divided by the number of cancer deaths. On an annual basis, the number of cancer deaths is reported in the Causes of Death Registry by Statistics Korea. The hospice utilization rate among cancer patients was 22.9% (2018), 24.3% (2019), and 23.0% (2020), respectively. The number of hospital beds assigned for HPC per 1,000,000 people is also computed for each administrative district using the population size reported by Statistics Korea (Population and Housing Census). As of 2020, the number of hospital beds assigned for HPC was 30 per 1,000,000 people in Korea. The province with the highest number was Jeonbuk (54 hospital beds for HPC per 1,000,000 people) and the lowest was Jeju (13 hospital beds for HPC per 1,000,000 people). In the capital city area (Seoul, Gyeonggi, and Incheon), the number of hospital beds for HPC per 1,000,000 people was around 27 to 30 (Figure 2).

## STRENGTHS AND WEAKNESSES

The most notable strength of the NHPC registry in Korea is its representativeness, which derives from collecting nationwide data on terminally ill cancer patients receiving HPC. The NHC has been providing reliable statistics on the current status of HPC in Korea. In North America and Europe, nationwide databases on HPC are rarely reported or limited to certain population groups [10]. For instance, the Center for Medicare and Medicaid Services (CMS) of the United States provides a hospice care data archive and releases relevant statistics [11]. The CMS database on hospice comprises elderly (65 years or older) Medicare beneficiaries [12]. Thus, the reports on Medicare patients enrolled in hospice programs are limited to a certain age group and insurance type in the United States [13]. Additional strengths include the high validity of the cancer diagnosis codes and the rigorous quality check procedures for constructing the database and the ability to compute national statistics on HPC combined with other sources of information provided by Statistics Korea without any data linkage (i.e., the hospice utilization rate and the number of hospital beds assigned for HPC can be calculated without linkage to the Death Registry or the Population and Housing Census).

Some limitations of the NHPC database should also be noted. The NHPC registry only enrolls terminally ill cancer patients who received inpatient hospice care. Thus, information on a control group (e.g., a similar group of terminally ill cancer patients admitted to the intensive care unit in the same hospital or elsewhere) is unknown, and this resource cannot be used to compare sociodemographic and clinical characteristics or EoL outcomes to those who received HPC [14]. Additionally, marital status and religion are self-reported (either by a patient or his or her legal guardian) without further validation. However, such information is usually only self-reported in other well-established population-based registries, such as the Community Health Survey and the Nurses’ Health Study [15,16].

## DATA ACCESSIBILITY

The National Hospice Center developed a website for releasing national HPC statistics in Korea (https://www.hospice.go.kr). Currently, a part of the National Hospice and Palliative Care database is available from the Public Data Portal (https://www.data.go.kr) managed by the Ministry of the Interior and Safety. The National Hospice Center regularly updates this information after a careful and comprehensive review.

## CONCLUSION

To produce national statistics of terminally ill cancer patients receiving inpatient hospice care, the NHPC registry was implemented. This registry and collects nationwide data in Korea on a regular basis that undergo extensive quality checks. The NHPC database consists of information on patients’ socio-demographic and clinical characteristics. The NHPC database is often used to address the current status of inpatient hospice care and serves as an important part of the real-world evidence associated with terminally ill cancer patients for policy-makers and other stakeholders.

## Figures and Tables

**Table 1.** Components of the National Hospice and Palliative Care database in Korea

Dataset	Variables
Patient socio-demographics	Basic information (name, sex, and resident registration number, location of residence), insurance type, marital status, religion, information on the primary caregiver^1^
Initial assessment of the patient	Information on terminal illness^2^, diagnosis date, KCD-6 codes, patient‘s awareness, clinical evidence for the diagnosis, level of consciousness, date of hospitalization, patient status upon hospital admission, pain scale^3^, date of discharge, reason for discharge
Hospice care duration	Duration of inpatient hospice care

KCD-6, Korean Standard Classification of Diseases-6.

1 Legally defined relationship (between the patient and caregiver) and whether the patient is living together with the primary caregiver or not.

2 Defined as an end-stage illness that is legally eligible for hospice care (cancer for inpatient hospice care).

3 Assessed at the time of the initial admission to the hospice ward.

**Table 2.** Total number of newly registered terminally ill cancer patients and medical institutions enrolled in the National Hospice and Palliative Care registry in Korea, 2018-2020

Category	2018	2019	2020
Total no. of patients^1^	15,859	16,798	15,254
Medical institutions^2^	-	-	-
Total no.	98	100	97
No. of beds^3^	1,542	1,577	1,546

1 Patients who were newly registered for inpatient hospice care.

2 Medical institutions (hospitals) specialized in hospice as designated by the Ministry of Health and Welfare.

3 Hospital beds assigned for inpatient hospice care (as of the last calendar date in each year).

**Table 3.** Socio-demographic characteristics of newly registered patients in the National Hospice Registration System of Korea, 2018-2020

Variable	2018	2019	2020
No. of patients	15,859	16,798	15,254
Age (yr)			
<39	277 (1.7)	256 (1.5)	233 (1.5)
40-49	867 (5.5)	878 (5.2)	744 (4.9)
50-59	2,319 (14.6)	2,484 (14.8)	2,095 (13.7)
60-69	3,710 (23.4)	3,941 (23.5)	3,775 (24.7)
≥70	8,686 (54.8)	9,239 (55.0)	8,407 (55.1)
Sex			
Male	9,250 (58.3)	9,766 (58.2)	8,929 (58.5)
Female	6,609 (41.7)	7,032 (41.8)	6,325 (41.5)
Location of residence			
Capital area	7,506 (47.3)	7,773 (46.2)	6,865 (45.0)
Metropolitan	3,384 (21.3)	3,655 (21.8)	3,715 (24.4)
Rural (city/town)	4,969 (31.4)	5,370 (32.0)	4,674 (30.6)
Insurance type			
NHIS^1^	14,274 (90.0)	15,137 (90.1)	13,766 (90.2)
Medical Aid	1,383 (8.7)	1,485 (8.8)	1,354 (8.9)
Other	202 (1.3)	176 (1.0)	134 (0.9)
Marital status			
Married	10,391 (65.5)	10,834 (64.5)	9,909 (65.0)
Bereaved	3,454 (21.8)	3,666 (21.8)	3,225 (21.1)
Divorced	1,175 (7.4)	1,343 (8.0)	1,307 (8.6)
Single	694 (4.4)	815 (4.9)	702 (4.6)
Separation	145 (0.9)	140 (0.8)	111 (0.7)
Religion			
Protestantism	4,178 (26.3)	4,487 (26.7)	3,746 (24.6)
Buddhism	2,835 (17.9)	2,851 (17.0)	2,713 (17.8)
Catholicism	2,047 (12.9)	2,264 (13.5)	1,842 (12.1)
Others	234 (1.5)	273 (1.6)	245 (1.6)
None	6,565 (41.4)	6,923 (41.2)	6,708 (44.0)
Primary caregiver			
Family	15,153 (95.5)	15,949 (94.9)	14,066 (92.2)
Others	706 (4.5)	849 (5.1)	1,188 (7.8)

Values are presented as number (%). NHIS, National Health Insurance Service.

1 Covered by the NHIS, including employee-insured and self-employed insured.

**Table 4.** Clinical characteristics of the newly registered patients receiving inpatient hospice care in the National Hospice Registration System of Korea, 2018-2020

Variable	2018	2019	2020
No. of patients	15,859	16,798	15,254
Cancer types			
Lung	2,482 (15.7)	2,943 (17.5)	2,849 (18.7)
Liver	1,679 (10.6)	1,961 (11.7)	1,760 (11.5)
Colorectal	1,627 (10.3)	1,928 (11.5)	1,713 (11.2)
Pancreas	1,485 (9.4)	1,814 (10.8)	1,674 (11.0)
Gastric	1,394 (8.8)	1,701 (10.1)	1,508 (9.9)
Others^1^	7,192 (45.3)	6,451 (38.4)	5,750 (37.7)
Awareness of terminal illness (patients)	12,940 (81.6)	13,930 (82.9)	13,045 (85.5)
Level of consciousness			
Alert	11,814 (74.5)	12,412 (73.9)	11,002 (72.1)
Drowsy	2,910 (18.3)	3,314 (19.7)	3,147 (20.6)
Stuporous	1,009 (6.4)	960 (5.7)	985 (6.5)
Comatose	126 (0.8)	112 (0.7)	120 (0.8)
Pain assessment at the time of initial registration^2^			
0 (none)	1,075 (11.2)	914 (9.4)	697 (8.1)
1-3 (mild)	4,910 (51.0)	5,090 (52.5)	4,570 (53.2)
4-6 (moderate)	2,638 (27.4)	2,790 (28.8)	2,442 (28.4)
7-10 (severe)	1,001 (10.4)	910 (9.4)	875 (10.2)
Duration of hospice care, days (median, IQR)	15 (6-31)	14 (6-30)	14 (6-30)

Values are presented as number (%). IQR, interquartile range.

1 Cancer types other than the ones listed above.

2 Pain assessment was based on a visual analogue scale determined by the medical staff.
